# Biomechanical Behavior of a Variable Angle Locked Tibiotalocalcaneal Construct

**DOI:** 10.3390/bioengineering7010027

**Published:** 2020-03-14

**Authors:** Farah Hamandi, Gerard Simon, Richard Laughlin, Tarun Goswami

**Affiliations:** 1 Department of Biomedical, Industrial and Human Factors Engineering, Wright State University, Dayton, OH 45435, USA; hamandi.3@wright.edu; 2 Air Force Research Laboratory, Materials and Manufacturing Technology Directorate, Structural Materials Division, Composites Branch, Dayton, OH 45433, USA; gerard.simon.2@us.af.mil; 3 Department of Orthopedic Surgery, Sports Medicine and Rehabilitation, Wright State University, Dayton, OH 45435, USA; richard.laughlin@wright.edu

**Keywords:** locking plate, finite element, nonlocking screw, locking screw, pitting, crack

## Abstract

This paper examines the mechanics of the tibiotalocalcaneal construct made with a PHILOS plating system. A failed device consisting of the LCP plate and cortical, locking, and cannulated screws was used to perform the analysis. Visual, microstructure, and fractographic examinations were carried out to characterize the fracture surface topology. These examinations revealed the presence of surface scratching, inclusions, discoloration, corrosion pits, beach marks, and cleavage and striations on the fracture surface. Further examination of the material crystallography and texture revealed an interaction of S, Ni, and Mo-based inclusions that may have raised pitting susceptibility of the device made with Stainless Steel 316L. These features suggest that the device underwent damage by pitting the corrosion-fatigue mechanism and overloading towards the end to fail the plate and screws in two or more components. The screws failed via conjoint bending and torsion fatigue mechanisms. Computer simulations of variable angle locking screws were performed in this paper. The material of construction of the device was governed by ASTM F138-8 or its ISO equivalent 5832 and exhibited inconsistencies in chemistry and hardness requirements. The failure conditions were matched in finite element modeling and those boundary conditions discussed in this paper.

## 1. Introduction

The locking compression plates in fractures fixation represents a major improvement especially in elderly patients [1]. However, the density of the bone decreases as individuals get older and that leads to complications and difficulties during fracture treatment [2]. Despite that, locking plates are mostly used with multiple fracture cases to optimize the alignment of the fracture area, reduce the gap between the bones, and provide maximum stability [3]. Conventional plating requires higher screw torque than the locking plates to provide the same amount of stability. This high torque can lead to screw loosening especially in osteoporotic bones and that causes an increase in the gap at the fracture area and failure of the fixation device(s) [4]. On the other hand, the locking plate provides more stability than the conventional plate. As the screws are implanted in fixed angles, there is no loosening between the plate and the bone that provides more stability to the fracture [5]. Additionally, having the screws in a fixed angle within the plate assists in equivalent stress distribution across the plate [6]. This advantage makes the locking plate a preferred choice when working on multiple fracture cases and bone with osteoporosis. In addition, the minimum insertion torque for bone fixation is the other advantage of the locking plates that assists in reducing postoperative complications [7]. Mechanical stability at the fracture location determines the healing type [8]. Primary healing occurs in the fracture area in cyclic compression. The primary fracture healing achieved via a locking plate allows bone cell formation [9], however, secondary fracture healing occurs via bridge fixation. Bridge fixation assists in the production of cartilage and bone produced in a similar way to the embryologic growth from the cartilage [9]. The other important advantage of the locking plate over the conventional plate is that the conventional plate depends on the friction between the bone and the plate only to provide the fixation, while the locking plate depends on locking the screw heads into the plate by the threaded holes. Having the plate and the screws locked together can be very beneficial for fracture fixation regardless of bone quality [10]. The limited or no motion at the fracture area provided by the locking plate keeps the fracture gap fixed at the area beneath the plate and allows micromotion at the far cortex [11]. Fracture gap (*FG*) strain can be expressed in the following relation:

(1) $$FG_{strain}=FG_{\Delta L}/FG_{L}$$

Additionally, it is important to know that fracture healing and strain have an inverse relationship. Less fracture gap strain might lead to primary fracture healing [11]. Perren [12] discussed that if $FG_{strain}$ is higher than 10% then no healing will occur at the fracture area. The length of the plate and the number of screws used have a significant effect on stress distribution across the plate. Studies exhibited that using a longer plate with widespread screws can reduce the pullout forces on the screws [11]. Conversely, spreading the screws would lead to decreasing the stiffness of the construct causing movement at the fracture region.

Stainless Steel 316L is one of the preferred materials to construct internal fixation devices. Strength, ductility, biocompatibility, and costs favor their use [13]. On the other hand, Shahryari and Omanovic [14] showed that one of the problems with stainless steel is pitting corrosion that associates with inclusions and affects device stability and leads to failure. Additionally, the in vivo environment enhances the pitting susceptibility [15]. Goswami and Hoeppner [16] discussed the transition of the pit(s) to a crack. This transition involves a six-stage mechanism: (1) incubation pit nucleation due to the mechanical process, (2) damage accumulation at a local point as a result of environment, and (3) microstructure interaction, (4) pit growth, (5) the transition from pit to fatigue crack growth, and finally (6) corrosion fatigue crack propagation. Additionally, they discussed the effect of the pit aspect ratio on the stress intensity factor and pitting corrosion, where having an aspect ratio (>1) would cause a pit to transition to a crack, which may propagate by corrosion fatigue mechanisms [16].

**Figure 1:**
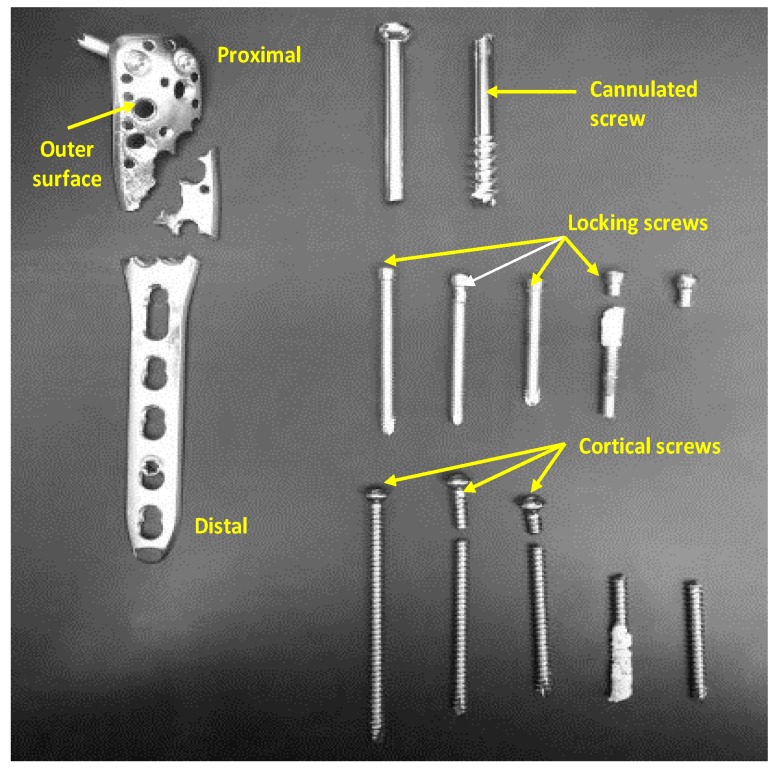
PHILOS Plating System failed parts**.**

The analysis of the tibiotalocalcaneal construct made with the PHILOS plating system (Figure 1) has been discussed in previous efforts; the plate and the screws were investigated separately [17,18]. This paper further elucidates the quantitative topography of microstructure and texture, damage mechanisms such as pitting and computational evaluations of loading and resulting stresses. In addition, we hypothesize that the angle of inserting the screws in the plate and the distance of the screw from the loading surface might affect the stress distribution and total displacement of the PHILOS plate, therefore the biomechanics of variable angle locking screws construct was performed with representative models investigating thirteen different insertion angles (0°, 5°, 10°, 15°, 20°, 30°, 40°, −5°, −10°, −15°, −20°, −30°, and −40°) and three different positions. The results from finite element analysis were used to develop the fatigue analysis using the traditional Paris equation and attempts made to interpret the crack nucleation phase controlled by the pit aspect ratio and crack propagation life estimated.

## 2. Materials and Methods

### 2.1. Material Conformity

To ensure that the plate and the screws have met the metallurgical requirements of ASTM standards F138-03 and F139-03 and the ISO equivalent 5832 for 316L stainless steel medical application material [19], X-ray Energy Dispersive Spectroscopy (EDS) was performed using an Octane Super detector. EDAX TEAM software was used to estimate the weight percent peaks for the plate, locking, cancellous, and cortical screws. 

### 2.2. Microstructure Characterization

Electron Backscatter Diffraction (EBSD) was performed on Thermo Fisher Scientific's innovative microscopy (FEI XL-30) using an EDAX EBSD detector to determine grain size and orientation. The grain area (*A_g_*) and diameter (*d_g_*) were calculated for each grain by using the ImageJ program. Then the mean grain size ($\bar{d}$) was calculated using Equation (2).

(2) $$\bar{d}=\frac{\sum A_{g}}{\sum d_{g}},\;$$

### 2.3. Device Failure Analysis Strategy

**Figure 2:**
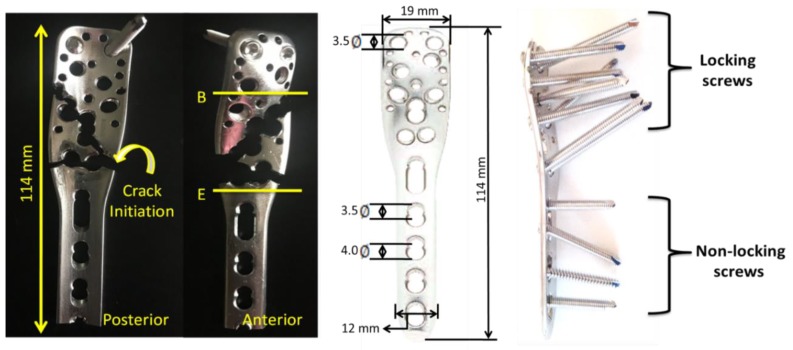
Anterior and posterior views of the PHILOS plate divided into six levels (**A**–**F**) showing the geometry, dimensions, and locking and nonlocking screw locations on the plate.

We divided the investigation of the PHILOS plate into six levels (A–F), as shown in Figure 2. Additionally, investigation on the screws was performed as well to understand the effect of screws on the failure of the PHILOS construct. The screws that have been investigated with SEM were CS1 (the proximal cortical screw), CS2 (the distal cortical screw), LC (the locking screw inserted at level C), and LE (the locking screw inserted at level E). Fractography was performed using FEI Quanta 650, at 15 kV, spot size 3, and aperture 30 µm. 

Striation spacing was determined from the fractographic examination on the surface of the CS1 screw. Then, the striation spacing was used to calculate the stress intensity factor (∆*K*) by using Bates and Clark equation [19] in its simplest form to estimate a range of stress intensity for the crack growth,

(3) $$\Delta K=E\sqrt{\frac{x}{6}}$$

where *x* is the average striation spacing, and *E* is the modulus of elasticity. *E*_316l stainless steel_ = 200 GPa [19].

### 2.4. Computational Modeling of Failure Mechanisms

In our previous study [18], finite element modeling was performed to simulate the loading conditions and stress distribution in the plate and the screws. In the current study, computational modeling was performed to predict failure mechanisms of the PHILOS construct (Section 2.4.1) and modeling variable angle screws (Section 2.4.2).

#### 2.4.1. Simulation of Fatigue Failure

**Figure 3:**
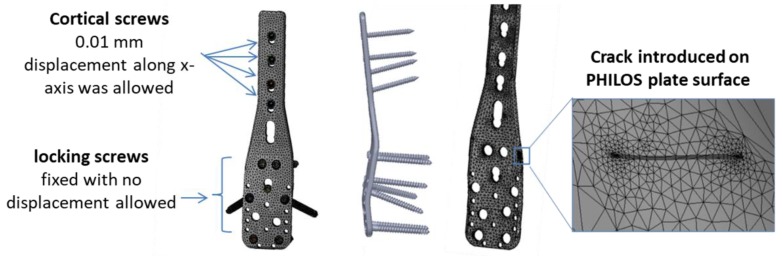
The PHILOS plate and crack meshing showing the locking and cortical nonlocking screw position and angle.

**Figure 4:**
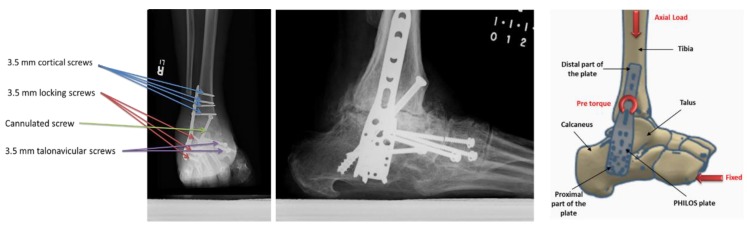
X-ray images of the PHILOS construct after two years showing the different types of screws used in fixation (cannulated and talonavicular screws were excluded from this study) (**left** and **middle**). The 3D model (**right**).

The stresses on the PHILOS construct vary continuously during the gait cycle resulting from activities of daily living such as walking or stairs climbing. This variation combined with bone friction against the plate and screws can initiate fatigue crack in the PHILOS construct. Inside the human body the pH value changes, so do temperature and load with activities, and these environments have been proven to cause corrosion of implants and fatigue cracking under cyclic loading. It is well known that fatigue precedes an incubation period which is both cycle and time-dependent. However, pit aspect ratios of sufficient size can form a crack which may propagate under steady-state condition or lead to tertiary crack propagation phase where abrupt failure may occur. To understand how the crack nucleates and propagates, a surface crack with different lengths was introduced in the modeling. In relation to the stress intensity factor present at the crack tip of the PHILOS plate under the same loading and boundary conditions used in Hamandi et al. [18], namely K_I_, those were determined from the FEA. One semi-elliptical crack was introduced on the surface of the plate at level E of the plate (Figure 3) simulating almost straight through-the-thickness cracks. Hence, cracks with an arc-length of 1 mm were introduced with four different aspect ratios (a/2c = 1, 0.8, 0.6, 0.4). The material properties were defined as elastic, homogeneous, and isotropic, with Young’s Modulus equal to 200 GPa and a Poisson’s ratio equal to 0.265. X-ray image of the PHILOS construct after two years, 3D model, boundary conditions, and loads are shown in Figure 4. The tetrahedral element type was applied to the model with approximately 94,000 nodes and 55,000 elements with the use of convergence tools in ANSYS Workbench 19.2 (ANSYS Inc., Canonsburg, PA, USA) with five percent convergence, and the meshing around the crack geometry was modified to a fine mesh.

#### 2.4.2. Modeling of Variable Angle Screws

**Figure 5:**
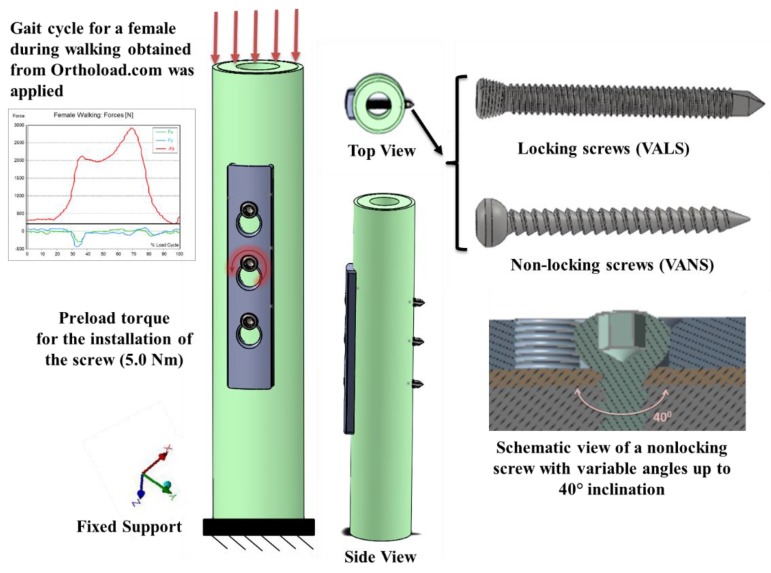
The 3D model of the bone, plate, and screws. Schematic view of a nonlocking screw with variable angles up to 40° inclination. Gait cycle for a female during walking obtained from Orthoload.com.

A new model was developed assuming that variable angles of screws and the distance from loading will have significant effects on stress distribution. The model comprised a hybrid plate with three holes, cortical and trabecular bones, and three screws, Figure 5. Two variable angle designs were developed, one with three locking screws (VALS) and one with three nonlocking screws (VANS). Additionally, the screws were mounted into 13 different angles (0°, 5°, 10°, 15°, 20°, 30°, 40°, −5°, −10°, −15°, −20°, −30°, −40°) with respect to the plate. We neglected the fracture size and any bone deformity, as occurred with subjects, and assumed an ideal scenario to simulate the effect of screws angles. We tried to overcome the limitations of previous models. The anisotropic material properties of the bone were considered, the gait cycle forces applied to observe the stress distribution, and the compression against the bone modeled differently between the locking and nonlocking screws. The walking gait cycle of the knee joint was obtained from the Orthoload website [20]. The material properties of the bone were obtained from [21], as an anisotropic representation of bone tissue was performed by calculating the elastic constants (Modulus of elasticity *E*, Poisson’s ratio *ν*, and shear modulus *G*) of cortical and trabecular parts with respect to radial, circumferential, and longitudinal directions. 

The following orthotropic relationships were used to calculate the elastic constants of the cortical part where the density (ρ) is equal to 1.071 g/cm^3^ [21]:

$$E_{\mathrm{radial}}=2314\rho^{1.57}$$

$$E_{\mathrm{circumferential}}=2314\rho^{1.57}$$

$$E_{\mathrm{longitudinal}\;}=2065\rho^{3.09}$$

While the following orthotropic relationships were used to calculate the elastic constants of the trabecular part where the density (ρ) is equal to 0.997 g/cm^3^ [21]:

$$E_{\mathrm{radial}}=1157\rho^{1.78}$$

$$E_{\mathrm{circumferential}}=1157\rho^{1.78}$$

$$E_{\mathrm{longitudinal}\;}=1904\rho^{1.64}$$

The elastic constants and elasticity tensor components were calculated (Table 1) and imported into the Ansys program for cortical and trabecular parts.

#### 2.4.3. Comparison with Experimental Work.

**Figure 6:**
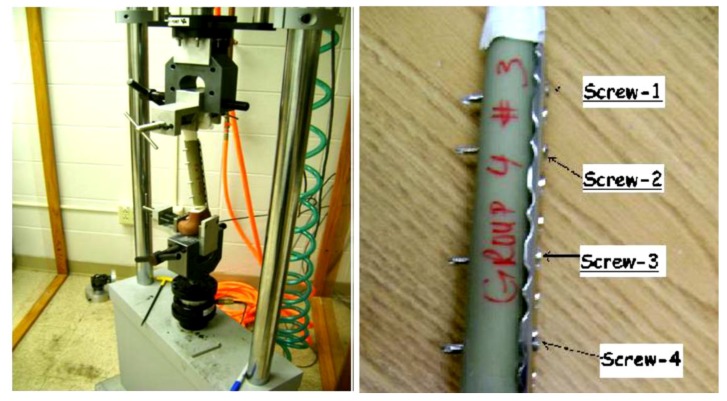
The synthetic bone with plate fixation in the EnduraTEC BOSE machine (**left**). The bone shaft showing the plate and screws [22] (**right**).

Experimental work performed at Miami Valley Hospital/Biomechanics Laboratory, Dayton, OH, included twenty synthetics bones [22] was compared with the proposed model, Figure 6. This comparison focuses on comparing the effect on the bone other than the plate and screws, as it was very important to investigate the computational simulation changes in bone stiffness with the experimental results to validate the simulation despite the design of the device.

## 3. Results

### 3.1. Material Conformity

**Figure A1:**
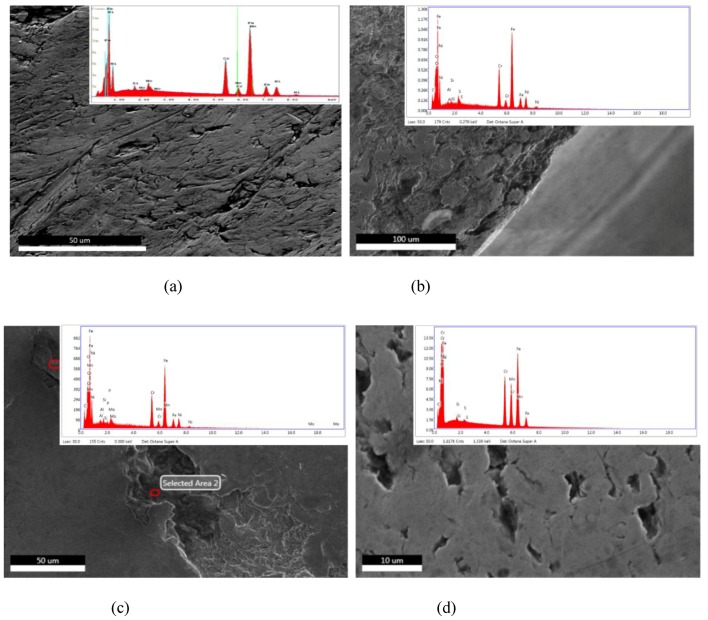
Energy-dispersive X-ray analysis shows the peaks of different element weight percent in (**a**) the plate, (**b**) cortical screw, (**c**) locking screw, and (**d**) cannulated screw.

Plate chemical composition was as follows (wt%): Cr (18.48), Ni (15.6), Mo (3.52), Mn (1.48), and Si (0.85). Additionally, the screw sample chemical composition was as follows (wt%): Cr (18.56), Ni (14.8), Mo (2.81), Mn (1.57), and Si (0.56). In general, EDS confirmed base material matches well with ASTM and ISO standard composition (see Appendix A Figure A1). Though, inclusions were identified on both the plate and the screws. The Rockwell hardness B-scale test was conducted from where the tensile strength of the plate derived [17,18]. The tests indicated that the hardness of the plate was with average (105.77) was 11% higher than the ASTM standard (95), while the tensile strength with average 987.3 MPa was 14% higher than the ASTM standard (868 MPa). Post-fracture data shows that there is a noticeable change in the hardness and tensile strength moving away from the fracture area. This may have occurred as a result of fatigue raising the hardness of the material near the fracture location. In addition, the tensile strength data shows some reduction in the elongation of the material.

### 3.2. Microstructure Characterization

**Figure 7:**
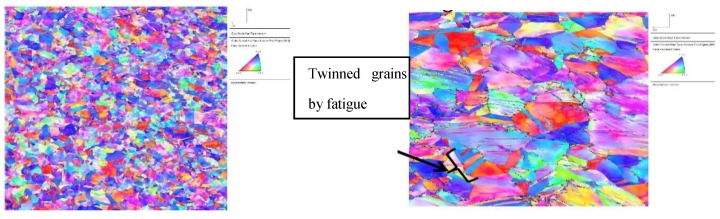
Electron backscatter diffraction (EBSD) grain map of the PHILOS Plate (**left**). Crystallographic characterization of plate microstructure inverse pole figure (**right**).

The map, Figure 7, shows a different orientation of the distribution of the grains on the surface of the material. ImageJ program was used to calculate the density percentages of different crystallographic orientations from the map of the PHILOS plate. The results showed that the density percentages were the highest at {1 1 0} plane and the least at the {1 1 1} plane. Literature showed that crystallographic orientation {1 1 1} is the least susceptible to pitting [23,24]. This means that having lower densities of these crystallographic orientations increases the susceptibility to pitting corrosion. Additionally, the grain map showed a random distribution. This random distribution of the grains indicates a weak texture with grain size distribution that acts as an additional factor to reduce the pitting corrosion resistance.

**Figure A2:**
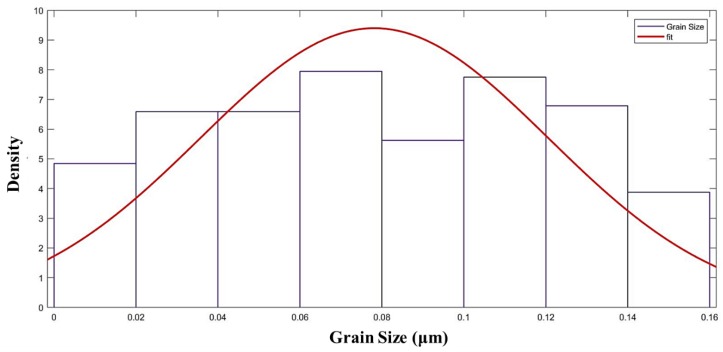
Density plot of grain size of 316l SS sample.

Figure A2 shows the frequency of grain size distribution for that sample area of 316L stainless steel PHILOS plate. Additionally, twinning was observed on the map, as shown in Figure 7, which had a considerable contribution to the displacement mechanism of the material. Further examination of the material revealed an interaction of S, Ni, and Mo-based inclusions that may have raised the pitting susceptibility of the device and suggest that the device underwent damage by pitting corrosion-fatigue mechanism and overloading towards the end to fail the plate and screws in two or more components, as discussed in Ina et al. [17].

### 3.3. Device Failure Analysis Strategy

**Figure 8:**
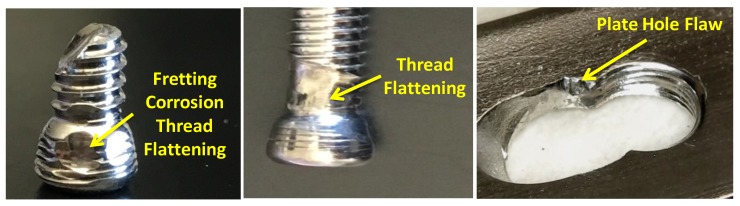
Thread flattening was observed on locking screws (**left, middle**) and plate hole flaw (**right**).

**Figure A3:**
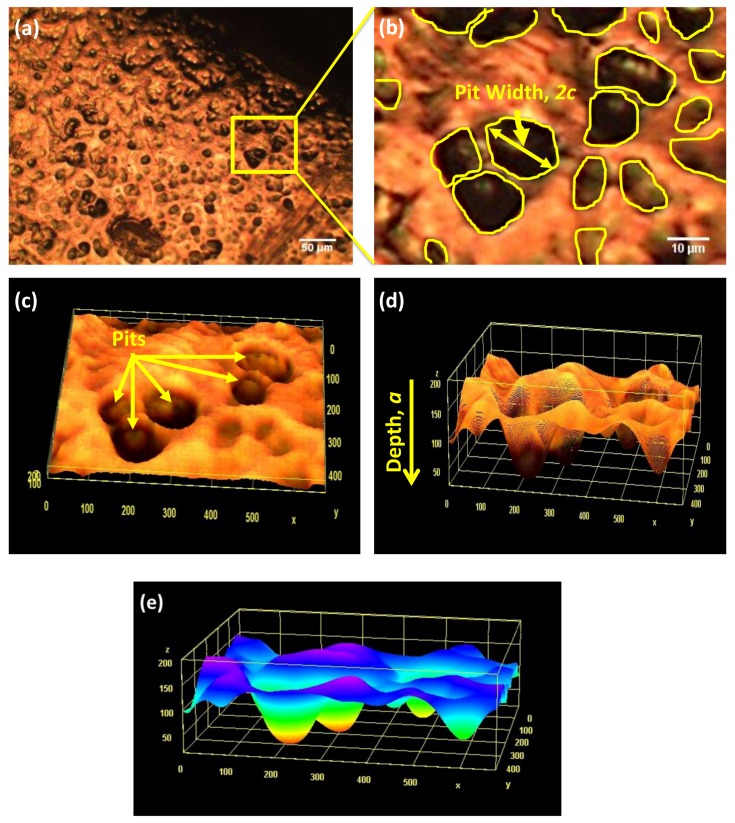
Pitting observed on the PHILOS plate. (**a**) High magnification image indicates pitting. (**b**) Higher magnification image showing pit boundaries. (**c**) 3D morphology; (**d**) 3D surface plot showing the depth of the pit. (**e**) The spectrum of the 3D surface plot (red represents the deeper part and blue represents the surface). The scale represents the number of pixels (10 pixels = 1 µm).

**Figure A4:**
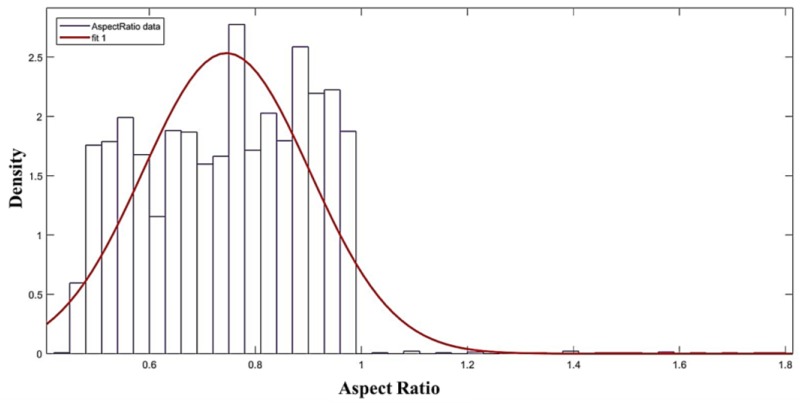
Density plot of pits aspect ratios ranges from (0.46–1.78).

**Figure A5:**
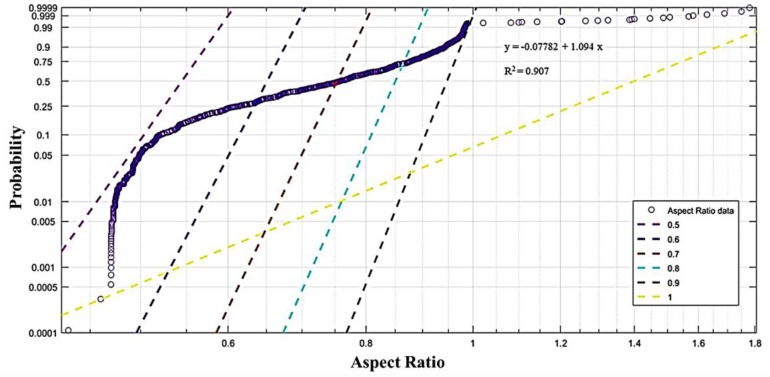
Probability plot of pits aspect ratio (Weibull, 95% CI).

Visual examination of the plate showed that it fractured into three pieces. One cannulated, three cortical, and three locking screws were fractured into two pieces. The images from the optical microscope showed scratches on both the plate and screws. Some of these scratches may have occurred during the removal of the device from the body or handling of the devices. Thread flattening was observed on locking screws and visible damage to the plate threading was observed as a result of in vivo micromotion, fretting between the screw and the bone and/or the plate, as shown in Figure 8. Pitting was observed also on the surface of the plate. ImageJ software was used to find pit aspect ratios (depth (a) over width (2c) ratio), as shown in Figure A3. The aspect ratio was calculated for each pit and it ranged from 0.46–1.78, and Figure A4 shows the density of pits aspect ratio distribution. Figure A5 plots the probability of failure with respect to pits aspect ratio and Weibull distribution with a 95% confidence interval, and the results are shown in Table 2. A probabilistic evaluation was performed on different aspect ratios (0.5, 0.6, 0.7, 0.8, 0.9, 1.0). It can be noticed that the highest distribution of the pit aspect ratio was within the range of 0.46 and 0.98.

The fractographic investigation illustrated that the PHILOS construct crack initiation has followed the six-stage mechanism proposed by Goswami and Hoeppner [16] where the transition from pit to crack started by incubation, pit nucleation, damage at a local point, pit growth, transition from pit to crack, and finally corrosion fatigue crack propagation. The fractography examination illustrated evidence that the fracture had started at the right side of the plate where we observed the highest number of pits and then progressed in diagonal and perpendicular directions that led to the failure of the plate into three pieces.

**Figure A6:**
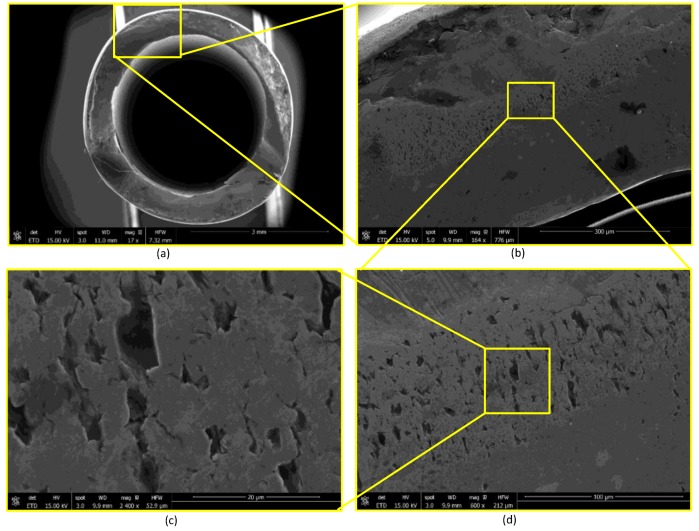
(**a**) Montage of SEM images showing the fracture surface of the cannulated screw, (**b**–**d**) dimples/overloading.

**Figure A7:**
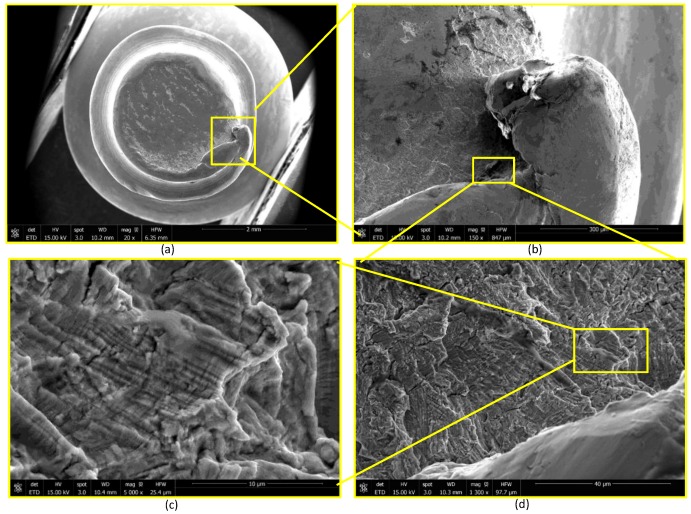
(**a**) Montage of SEM images showing fracture surface of the cortical screw (CS1), (**b**–**d**) striations, river lines perpendicular to striations. River lines point towards the initiation point (river lines run bottom left to top right).

**Figure A8:**
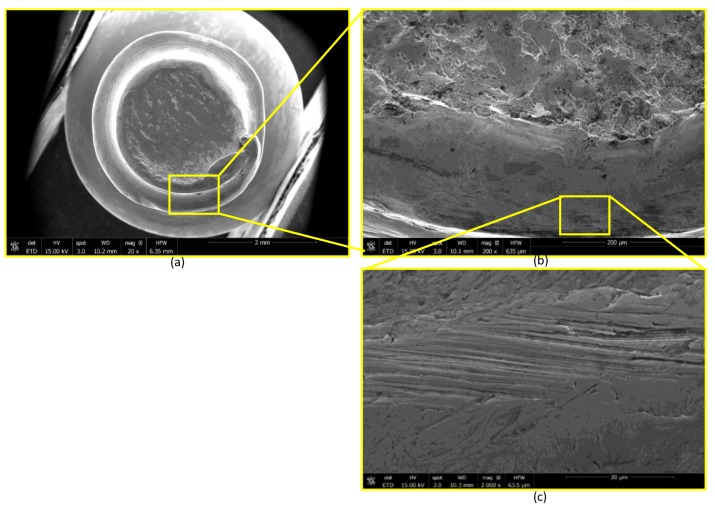
(**a**) Fracture surface of the cortical screw (CS1) and (**b**,**c**) mechanical damage caused by scratching the other side after failure.

**Figure A9:**
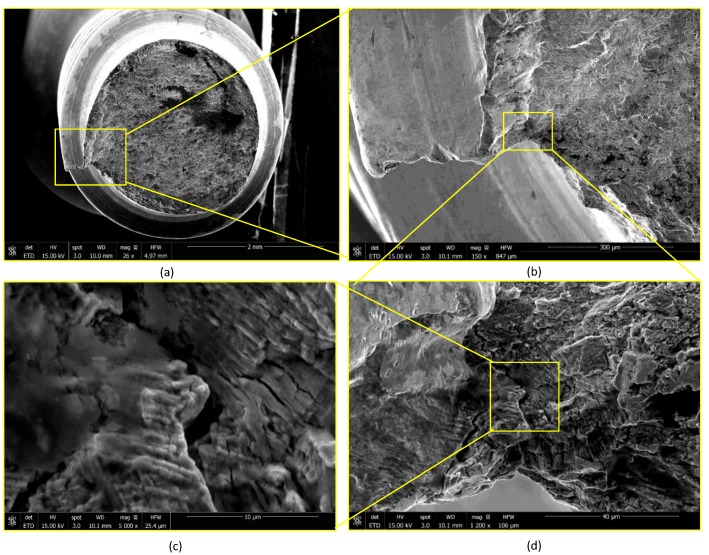
(**a**) Montage of SEM images showing the fracture surface of the cortical screw (CS4), (**b**–**d**) fatigue striations.

**Figure A10:**
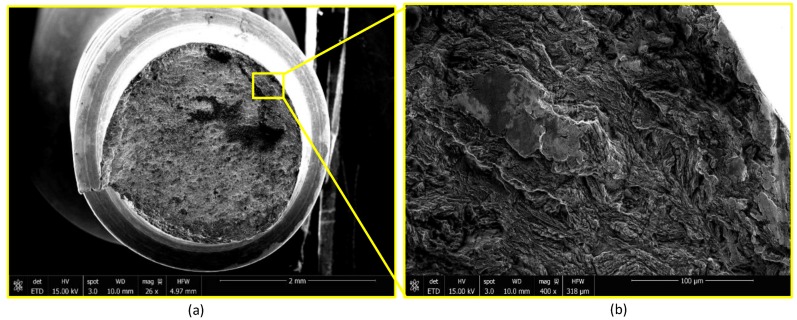
(**a**) Montage of SEM images showing the fracture surface of the cortical screw (CS4) (**b**) flattening from rubbing.

**Figure A11:**
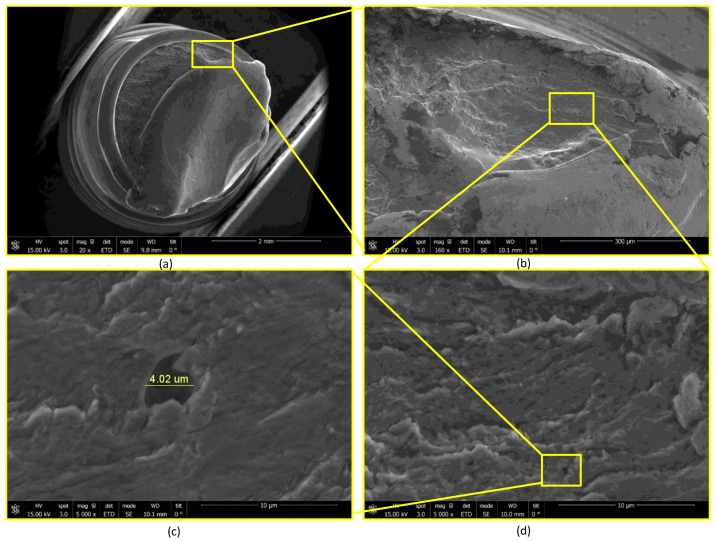
(**a**) Montage of SEM images showing fracture surface of locking screw (LC), (**b**–**d**) pore or site of inclusion pull out after fracture resulting in dimples and/or porosity.

**Figure A12:**
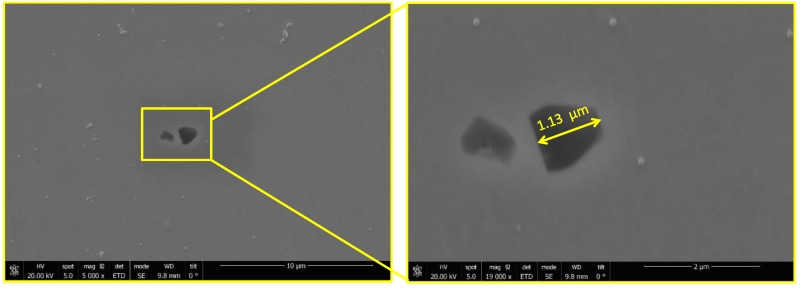
Evidence of Si-based inclusions, these inclusions have sharp points and could be crack initiators.

**Figure A13:**
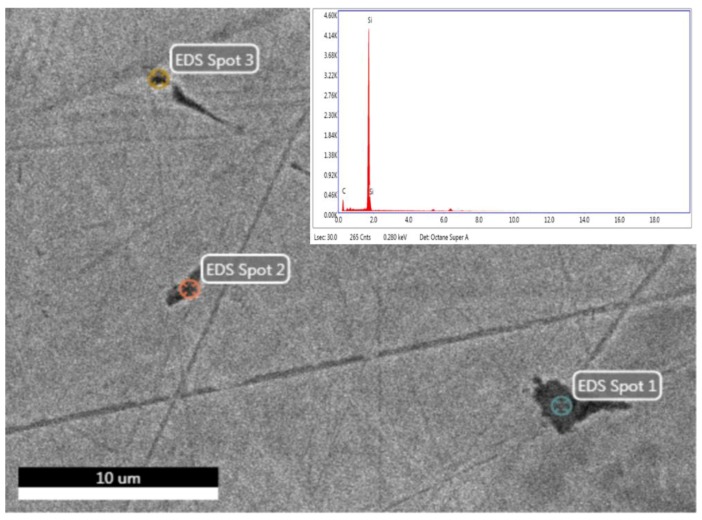
CS4 EDS showing Si-based inclusions.

**Figure A14:**
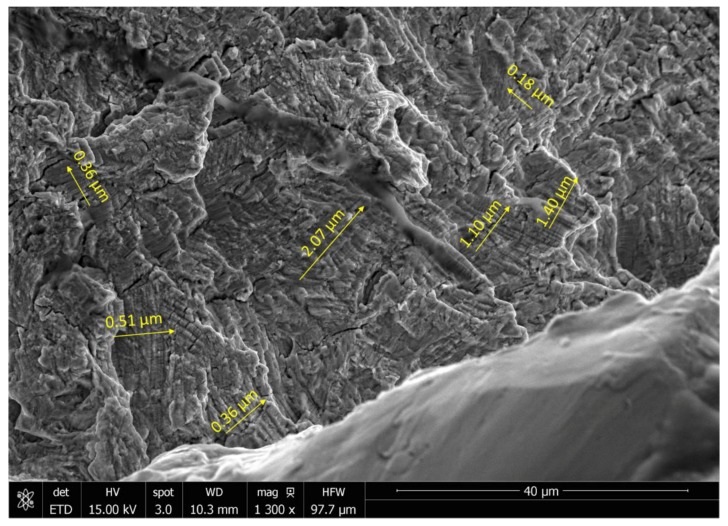
Counting the average striation spacing on the surface of the CS1 screw shows that the crack propagated from the bottom left to the top right.

**Figure A15:**
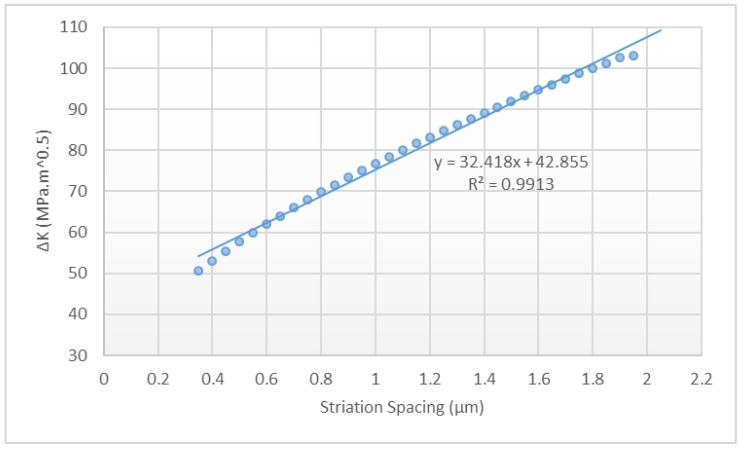
Stress intensity factor (∆K) vs. striation spacing.

**Figure A16:**
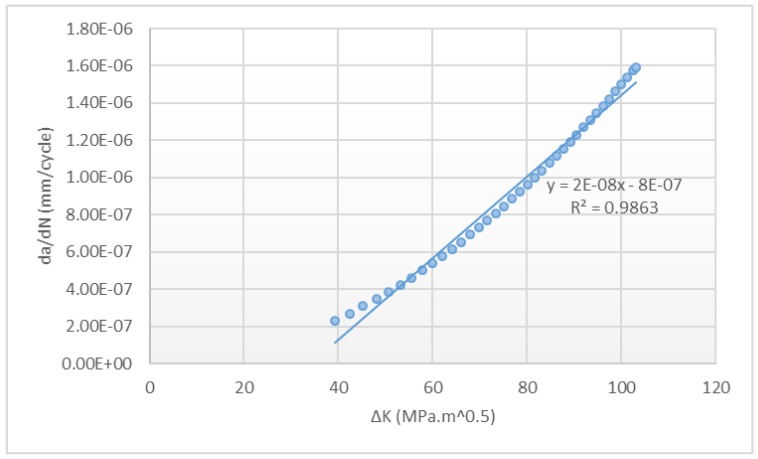
Stress intensity factor (∆K) vs. fatigue crack growth (da/dN) as estimated by the Bates and Clark equation.

It was observed that most pieces showed extensive post-fracture rubbing/displacement which obscures any obvious fracture information. However, striations were found in CS1 and CS4. Cannulated screw showed indications of intergranular cracking, as shown in Figure A6 and Figure A7. Additionally, the CS1 cortical screw showed striations and river lines perpendicular to striations, and river lines point towards the initiation point, as shown in Figure A8. In addition, some mechanical damage caused by scratching the other side after failure can be observed, as shown in Figure A9. CS4 cortical screw showed shiny marks indicative of fatigue damage and secondary cracks; though there was no strong indication of directionality, as shown in Figure A10. Flattening from rubbing is shown in Figure A11. LC locking screw showed a pore or site of inclusion pull out after fracture (Figure A12). Finally, Figure A13 and Figure A14 show evidence of Si-based inclusions. These have sharp points and could be crack initiators. In general, screws investigation illustrated inclusions, pitting, secondary cracks, and striations that may be an indication of device failure as a result of the corrosion-fatigue mechanism. However, upon linking with the screw-holes, a crack, perpendicular to loading direction also propagated resulting in multiple fractures. Additionally, striations presence at the bending areas illustrates that the screws failed via conjoint bending and torsion fatigue mechanisms, discussed in another study [24]. Striation spacing was determined from the fractographic examination on the surface of the CS1 screw, as shown in Figure A15. Figure A16 shows a linear trend with R^2^ = 0.99 between ΔK and striation spacing and $\frac{da}{dN}$, which means that the relationship is nearly linear.

The visual and fractographic examinations demonstrate that the crack started anteriorly then progressed in a diagonal direction, 45° to the loading direction, posteriorly. However, upon linking with the screw-holes, a crack, perpendicular to loading direction also propagated resulting in multiple fractures.

### 3.4. Computational Modeling of Failure Mechanisms

#### 3.4.1. Simulation of Fatigue Failure

**Figure 9:**
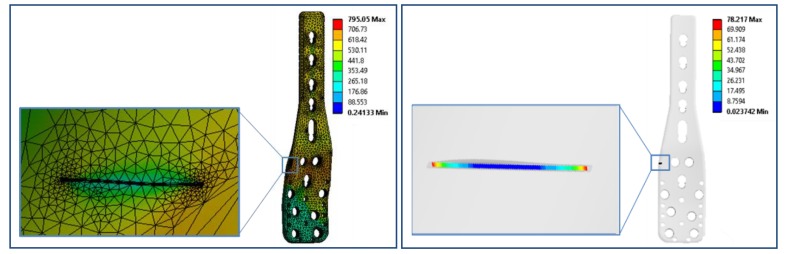
The von-Mises stresses in the 316L SS plate with screw design B, 2000 N axial load, 0.5 coefficient of friction between the screw and the plate, 0.1 mm cortical screw displacement, and 1 mm crack length (**left**). The stress intensity factor distribution across the crack for 0.6 aspect ratio (**right**).

The stress intensity factor was estimated at the tip of the crack nodes in the form of six contours. The contours were used to identify the regions of high and low von Mises stresses. It can be noticed that the maximum von Mises stresses are at levels B–E. Moreover, K_I_ values were determined for four different aspect ratios (0.4, 0.6, 0.8, and 1). The stress intensity factors, maximum von Mises stresses, and fracture toughness for each crack length are presented in Table 3. The average value of K_I_ was used for further analysis to calculate the stress needed to cause the final failure of the PHILOS construct (Section 3.4.4). Figure 9 illustrates the von Mises stress of the plate (795 MPa) with screw design B, 2000 N axial load, 0.5 coefficient of friction between the screw and the plate, 0.1 mm cortical screw displacement, and 1 aspect ratio. Fatigue limit at 10^7^ cycles (stress level below which fatigue does not happen) corresponds to 440 MPa maximum stress level [25]. As the quantitative analysis of pits and resulting aspect ratios are much higher, we need to explore the pit transition to pitting corrosion fatigue crack growth mechanisms and life of the PHILOS constructs controlled by corrosion fatigue crack propagation than the durability aspects of fatigue design.

#### 3.4.2. Modeling of Variable Angle Screws

**Figure 10:**
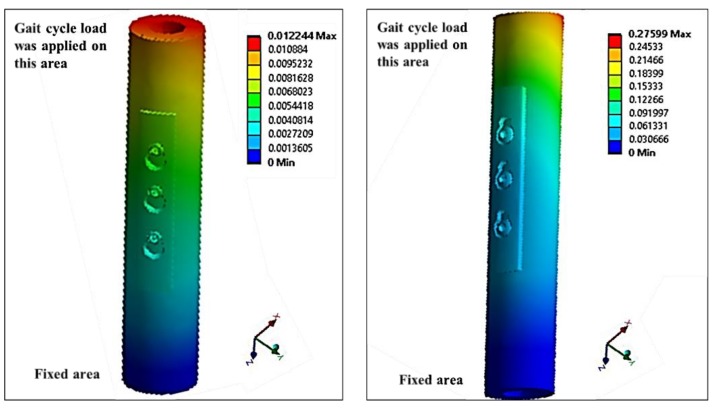
The total displacement (mm) in the 3D model of the plate with screws perpendicular to the plate. The variable angle locking screws (VALS) model (**left**) and the variable angle nonlockimg screws (VANS) model (**right**).

**Figure A17:**
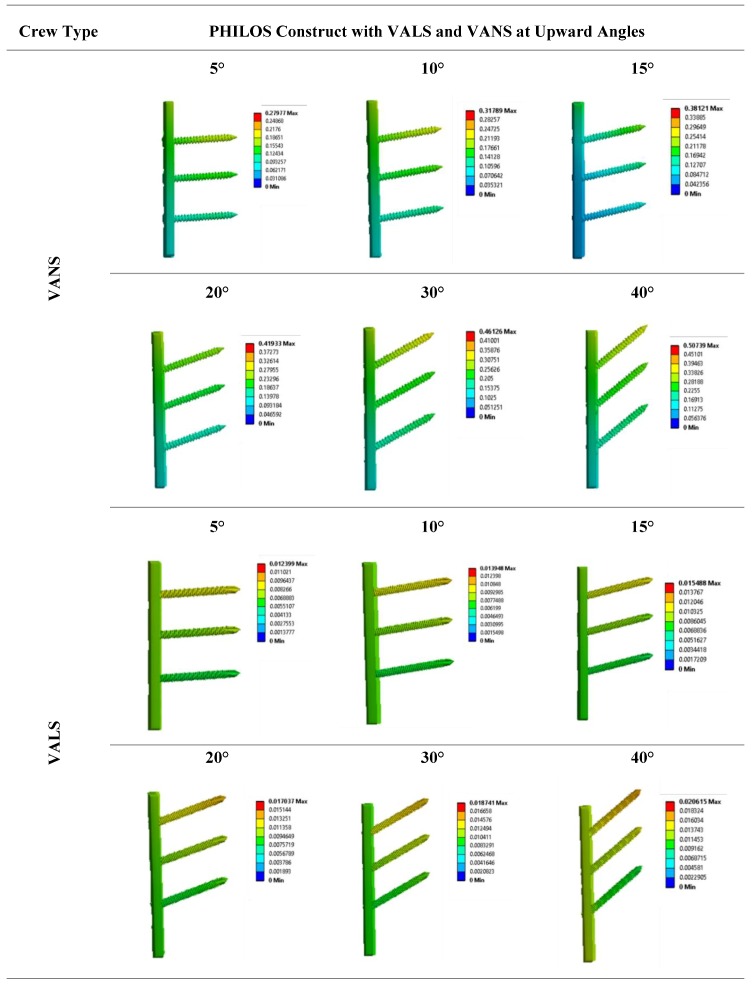
Total displacement (mm) of the PHILOS construct with VALS and VANS at upward angles (5°, 10°, 15°, 20°, 30°, 40°) to the neutral axis.

**Figure A18:**
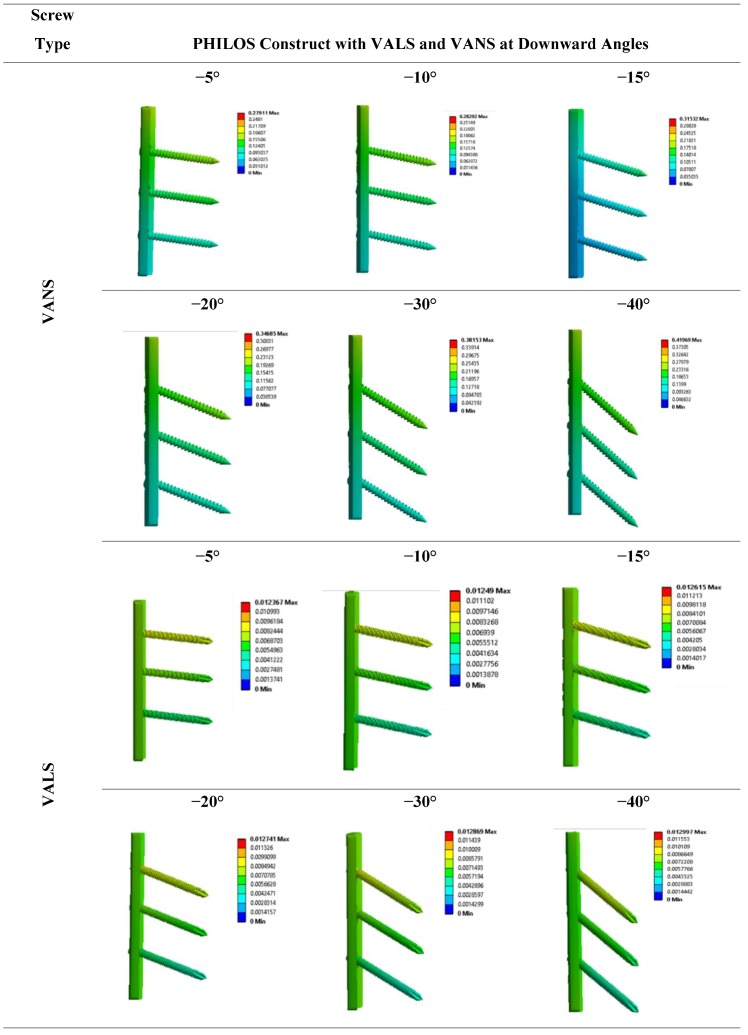
Total displacement (mm) of the PHILOS construct with VALS and VANS at downward angles (5°, 10°, 15°, 20°, 30°, 40°) to the neutral axis.

**Figure A19:**
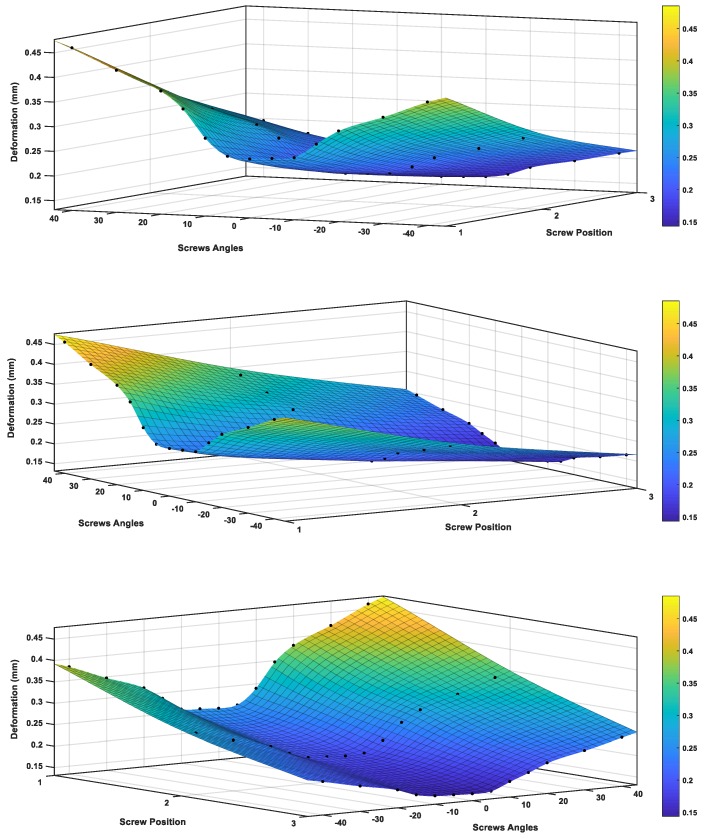
VANS displacement vs. screw angles and screw position.

**Figure A20:**
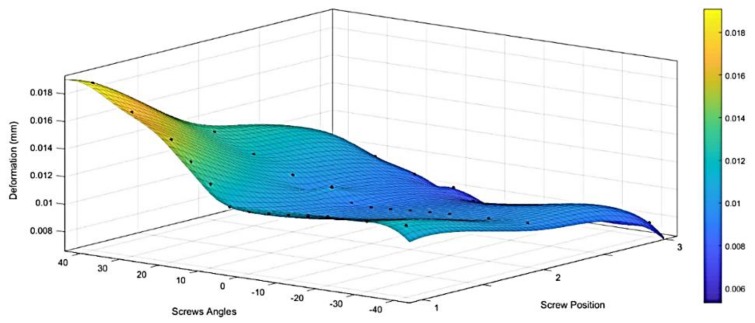
VALS displacement vs. screw angles and screw position.

Previous FEA results showed that the angle of the screw might have had an effect on increasing the stress distribution across the screws and the plate. The results of the 26 models (13 VALS & 13 VANS) illustrate that there is a significant difference between the screws at different levels, as shown in Figure 10 and Figure A17, Figure A18, Figure A19 and Figure A20. The total displacement of the plate with a perpendicular angle to the neutral axis (0°) is twenty times higher in VANS compared with VALS, shown in Figure 10. The stress and displacement are highest at screw one (0.25 mm) and least at screw three (0.15 mm). It can be noticed for VALS, the displacement of screw one is 21% higher than the second screw and 55% higher than screw three. While for VANS the difference is more noticeable to be 34% higher than the second screw and 70% higher than the third screw. Additionally, the stress and displacement increased significantly with the increase in the angle of the screws to be the highest at 40 degrees above the neutral axis (from the center of the screw hole). Figure A17 and Figure A18 show the total displacement of the PHILOS construct with VALS and VANS at different angles. VALS results show that the displacement is (0.01 mm) when the VALS is perpendicular to the plate and increased by 1%, 14%, 26%, 39%, 53%, 68% as the angle increased by 5°, 10°, 15°, 20°, 30°, and 40°, respectively. However, the displacement increased by 1%, 2%, 3%, 4%, 5%, and 6% with −5°, −10°, −15°, −20°, −30°, and −40°, respectively. VANS results show that the displacement is (0.25 mm) when the VALS is perpendicular to the plate and increased by 1%, 15%, 38%, 51%, 67%, and 83% with 5°, 10°, 15°, 20°, 30°, and 40° and an increase of 1%, 3%, 14%, 26%, 38%, and 52% with the negative angles of 5°, 10°, 15°, 20°, 30°, and 40°.

**Figure 11:**
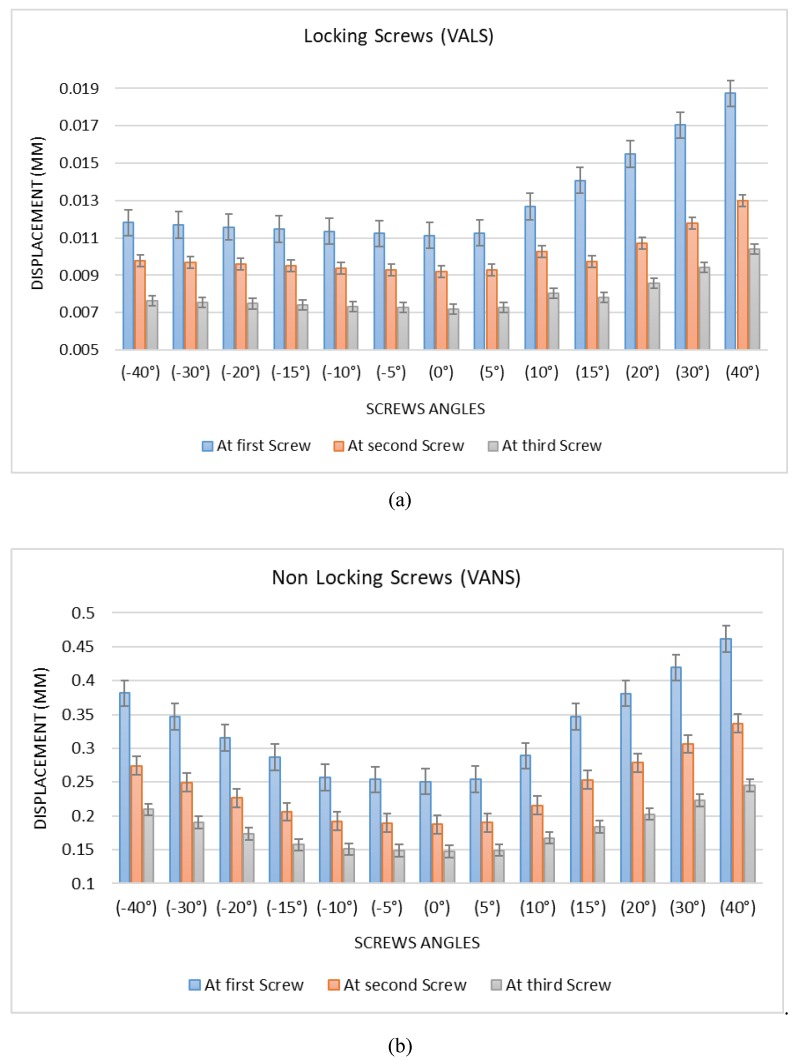
(**a**) VANS displacement. (**b**) VALS displacement.

Figure 11a shows that there is a distinct difference between the positive slope of the upward angles and downward angles of VALS, as the upward angles results have a higher positive slope and the displacement of VALS with 40° upward angle is (59%) higher than VALS with 40° downward angle. Similarly, Figure 11b shows that there is a distinct difference between the positive slope of the upward angles and downward angles of VANS, as the upward angles results have a higher positive slop and the displacement of VANS with 40° upward angle is (21%) higher than VANS with 40° downward angle. Furthermore, having the VANS in downward angles showed a significant increase when compared with the screws in perpendicular angles to the plate (52%), while there was no significant change in the VALS (6%).

The sensitivity plot (Figure A19) shows the relationship between the VANS angles and position used in the models and the total displacement results. Increasing the angles in upward or downward directions at screw one (close to the applied load) results in higher displacement (0.46 mm). However, as the screw position gets further away from the applied load and the VANS angle reaches 0°, then the displacement decreases as well (0.25 mm). On the other hand, the sensitivity plot (Figure A20) shows the relationship between the VALS angles and position used in the models and the total displacement results. Increasing the angles in the upward direction at screw one (close to the applied load) results in higher displacement (0.02 mm) with no noticeable increment in the downward direction. However, as the screw position gets further away from the applied load and the VALS angle decreases, then the displacement decreases (0.01 mm). The peak on the sensitivity plots for both VALS and VANS corresponds with the upward 40° angle at screw one.

#### 3.4.3. Comparison with Experimental Work

**Figure 12:**
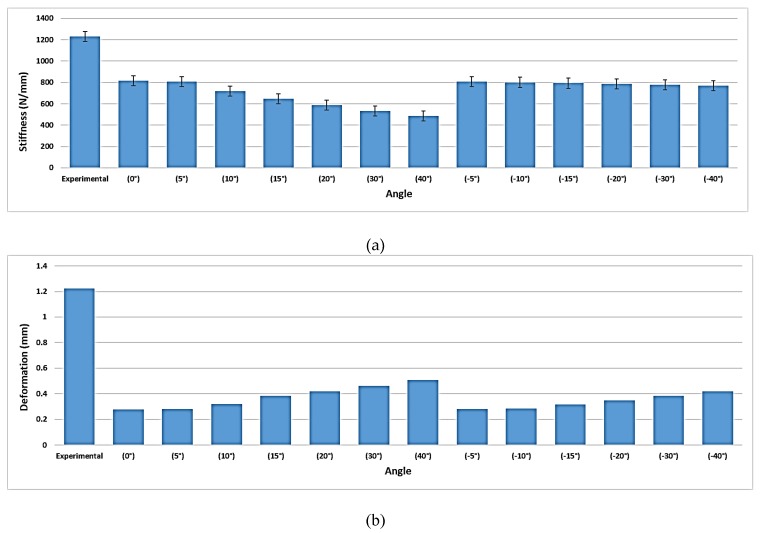
(**a**) The stiffness for the experimental work versus VALS FE results. (**b**) The displacement for the experimental work versus the VANS FE results.

Stiffness and displacement were measured for different screw combinations groups, as shown (see Appendix A Table A1). It can be noticed that displacement results are 52% higher in the experimental work than the finite element simulation. This may have been a result of the grip-to-grip change in length versus the change in length between two reference points in the simulations and the combined axial and torsion loads. Even though we notice such a change in length, the two results are acceptable with a degree of confidence. However, the stiffness results were in a good agreement with the experimental results. This establishes the applicability of finite element simulation in more complex designs, as shown in Figure 12. Figure 12a shows the highest stiffness when the screws are perpendicular to the plate (0°) and the stiffness decreases as the angle increases to be 40° upward. While there is no significant decline in the stiffness as the angle decreases in the negative side. Additionally, Figure 12b illustrates that there is a significant difference between the experimental displacement results (1.2 mm) and FE displacement results (0.5 mm). In addition, it can be noticed that the displacement increases as the angle upward or downward changed to be the highest at a 40° upward angle (0.5 mm).

#### 3.4.4. Fatigue Analysis of PHILOS Construct

To understand the failure mechanism, it is important to estimate the number of cycles to failure (*N_f_*). The X-ray images showed that the first screw failed after two years and the plate failed after six years. Assuming the 68 years old subject had limited activities with 4000 step/day [26] for 6 years, and to calculate the number of cycles that led to the failure of the PHILOS plate, we can multiply the number of cycles each day for one leg times the number of days before failure, as follows:

$$Screw\;N_{f}=2000\frac{cycle}{day}*\left(730\right)days=1,460,000\;cycle$$

$$Plate\;N_{f}=2000\frac{cycle}{day}*\left(2190\right)days=4,380,000\;cycle$$

As the cracking failed the plate, we need to invoke fracture mechanics concepts in our modeling methods. The life of the plate was controlled by crack nucleation via pitting corrosion documented and modeled in this paper. We are not able to comment on the time it may have taken to form a pit and subsequently growing it to become an engineering crack. Although it is important to estimate the crack initiation stresses (σ) for both the screws and the plate, a semi-log relationship is used to calculate the stress with respect to the number of cycles to failure that we calculated above.

$$\sigma_{max}=A+B\log N_{f}$$

To find *A* and *B*, the ultimate stress of 316 L stainless steel (860 MPa) and the lower limit of fatigue at 10^7^ cycles (440 MPa) were used, as follows

(4) 860 Mpa=A+Blog(0)

(5) $$440\;\mathrm{MPa}=A+B\log\left(10^{7}\right)$$

From that, *A* = 930 and *B* = −70. Therefore, the maximum stress needed to initiate the crack of the plate for 4,380,000 cycles to failure is 465 MPa. This means that 465 MPa is the maximum stress needed to initiate the fatigue crack of the plate.

Additionally, Paris law was used to calculate the growth of the fatigue crack. Where C and m are material parameters with the corrosion fatigue effect equal to 8.47 × 10^−11^ and 2.3, respectively [27].

Initial crack size is 0.4, 0.6, 0.8, 1.0. The final crack size is calculated from the equation of fracture toughness

(6) $$a_{f}=\frac{1}{\pi }{\left(\frac{K_{1c}}{\sigma }\right)}^{2}$$

By using the FE results in (Section 3.4.1), $a_{f}$ is 6.57 mm, 6.46 mm, 6.34 mm, and 6.32 mm for $a_{0}$ equals to 0.4, 0.6 mm, 0.8 mm, and 1.0 mm, respectively. From that, the fatigue cycles that are estimated to cause fracture ($N_{f}$) is estimated to be 2.54 × 10^4^ cycles to cause failure with the final crack size 6.5 mm.

Moreover, the stress intensity factor (*K_I_*) that was calculated in (Section 3.4.1) is used to calculate the stress (σ_f_) needed to cause the final failure of the plate. The Newman and Raju theoretical approach for semi-elliptical surface crack is used to find σ_f_ [28], as follows:

(7) $$k_{I}=\left(S_{t}+H_{s}\sigma_{f}\right)\sqrt{\pi \frac{a}{Q}}F_{s}\left(\frac{a}{c},\;\frac{a}{t},\;\frac{c}{b},\;\varphi \right)$$

where *S_t_, H_s_, Q, F_s_, t*, and φ are remote uniform tension stress, bending multiplier for a surface crack in a plate, shape factor for semi-elliptical crack, boundary-correction factor for a surface crack in a plate, thickness, and parametric angle of the ellipse, respectively.

(8) $$F_{s}=\left[M_{1}+M_{2}{\left(\frac{a}{t}\right)}^{2}+M_{3}{\left(\frac{a}{t}\right)}^{4}\right]gf_{\varphi }f_{w}$$

(9) $$M_{1}=1.13-0.09\left(\frac{a}{c}\right)$$

(10) $$M_{2}=-0.54-\frac{0.89}{0.2+\frac{a}{c}}$$

(11) $$M_{3}=0.5-\frac{1}{0.65+\frac{a}{c}}+14{\left(1-\frac{a}{c}\right)}^{24}$$

(12) $$g=1+\left[0.1+0.35{\left(\frac{a}{t}\right)}^{2}\right]{\left(1-\sin\;\varphi \right)}^{2}$$

(13) fφ=[(ac)2cos2φ+sin2φ]14

(14) fw=[sec(πc2bat)]12

(15) $$Q=1+1.464{\left(\frac{a}{c}\right)}^{1.65}$$

where *a* = 0.4, 0.6, 0.8, and 1 mm, *b* = 15 mm, *c* = 0.5 mm, *t* = 3 mm, *S_t_* = 0 MPa, $H_{s}$ = 0.098, 0 ≤ φ ≤π, φ = π/2 at the deepest point of the crack and φ = 0, π on the edges, K_I_ values were obtained from the finite element simulation. The calculation illustrated that σ_f_ needed to cause the final failure of the plate is 1003 MPa for 0.6 mm crack. This stress is significantly higher than the ultimate strength of 316 L stainless steel (860 MPa). This high stress indicates that overloading took place and led to a complete failure of the device, which supports the idea that the nonunion of the tibiotalocalcaneal fracture that was not documented until 18 months after surgery resulted in high loads on the screws primarily and caused overloading on the plate.

## 4. Discussion

This paper examines the biomechanical behavior of the VALS and conditions that may have failed a tibiotalocalcaneal construct made with SS 316L. The PHILOS device is used in humerus fracture fixation though recommended use also extended to the tibiotalocalcaneal joint examined in this paper. Visual examination of the plate illustrates that it fractured into three pieces and the screws pulled to fracture. One cannulated, three cortical, and three locking screws failed into two pieces. Fractography elucidates extensive post-fracture rubbing that affected the visualization of the obvious fracture information. The investigation illustrates that the crack initiation in the PHILOS construct followed the six-stage mechanism proposed by Goswami and Hoeppner [16] where the transition from pit to crack started by incubation, pit nucleation, damage at a local point, pit growth, transition from pit to crack, and finally corrosion fatigue crack propagation. The fractographic examination proved that the fracture started at the right side of the plate and progressed at 45° and at 90° directions that led to the failure of the plate into three pieces. However, we are unable to describe these two independent crack growth mechanisms sequentially and whether or not the two cracks failed the plate instantaneously. In addition, inclusions were identified on both the plate and the screws, and evidence of Si-based inclusions with sharp points were observed that could have been a crack initiator. On the other hand, striations were found in CS1 and CS4. Cannulated screw showed indications of intergranular cracking. Additionally, the CS1 cortical screw showed river line patterns perpendicular to striations. CS4 cortical screw showed cracks and flattening from rubbing. However, there was no strong indication of directionality. In general, screws consisted of inclusions, pitting, secondary cracks, and striations that may be an indication of device failure as a result of the corrosion-fatigue mechanism.

### 4.1. Microstructure Characterization

The grain size and orientation map show different orientations on the surface of the material. This random distribution of the grains indicates a weak texture. Twinning was observed on the microstructure, which had a considerable contribution to the displacement mechanism of the material. However, it is also possible that the material contained twins. Shahryari et al. [23] linked the orientation of the grains and pitting behavior of 316L stainless steel material. In their study, 316LVM SS samples were tested experimentally by using the orientation imaging microscopy method. Their results showed that the density percentages were the highest at the {1 1 0} plane and the least at the {1 1 1} plane. This means that having lower densities of these crystallographic orientations increases the susceptibility to pitting corrosion. Additionally, pitting was observed also on the surface of the plate, and the aspect ratio was calculated for each pit and it ranged from 0.46–1.78. Once a pit reaches a critical aspect ratio, it transitions to a crack. As it propagates the local stress-strain distribution at the wake of the crack determines how it will grow. However, the pits away from the crack tip, on the free surfaces, will continue to grow as it is a time-dependent process. As a result, pits on the plate edges, from where the crack started, kept growing during the entire duration the device was in vivo resulting in higher aspect ratios. Furthermore, the material revealed an interaction of S, Ni, and Mo-based inclusions and may have raised pitting susceptibility of the device, which suggests that the device underwent damage by pitting the corrosion-fatigue mechanism and overloading towards the end to fail the plate and screws in two or more components.

### 4.2. Computational Simulation

The dynamic computational modeling of the PHILOS plate developed 795.05 MPa at 2000 N axial load as a result of body weight and activity levels, which is higher than the fatigue limit at 10^7^ cycles (440 MPa), meaning daily activity such as walking was high enough to start the initiation of the crack propagation. VALS and VANS were computationally modeled to support our assumption that variable angles of screws and the distance from loading have significant effects on plate internal fixation. The stress vs. displacement behavior found to be dependent on where the screws are located on the plate. The closer the screws were to the level where the forces applied those were the regions of higher stress distribution. The FE results of the VALS and VANS models illustrate that there is a significant difference between the screws at different levels. The stress and displacement were highest at the first screw and least at the last screw. In addition, the VALS displacement at the screw one was 21% higher than the second screw and 55% higher than screw three, and it was more noticeable for VANS to be 34% higher than the second screw and 70% higher than the third screw.

The total displacement of the plate with (0°) angle was twenty times higher in VANS compared with VALS. This significant difference occurred as a result of fixing the locking screw heads to the plate in VALS that reduced the amount of allowed displacement, while the displacement was allowed for the nonlocking screws. Additionally, the stress and displacement significantly increased with the increase in the angle of the screws to be the highest at 40 degrees above the neutral axis. FEA of VALS showed that the displacement is (0.01 mm) when the VALS is perpendicular to the plate and increased by 1% as the angle increased 5° and increased by 68% as the angle increased 40°. On the other hand, the displacement increased by only 6% as the angle decreased to 40°. This means that having the VALS in perpendicular or downward angles are more preferred than upward angles of more than 15°. The FEA of VANS showed that the displacement is (0.25 mm) when the VANS is perpendicular to the plate and increased by 1% as the angle increased 5°, increased by 51% as the angle increased 20°, and increased by 83% as the angle increased 40°. Similarly, the displacement increased by 52% as the angle decreased to 40°. This means that having VANS in upward or downward angles more than 15° is not preferred. In general, there is a distinct difference between the positive slope of the upward angles and downward angles of VALS, as the upward angles results have a higher positive slope and the displacement of VALS with 40° upward angle is (59%) higher than VALS with 40° downward angle. Similarly, there is a distinct difference between the positive slope of the upward angles and downward angles of VANS, as the upward angle results have a higher positive slope and the displacement of VANS with 40° upward angle is (21%) higher than VANS with a 40° downward angle. Furthermore, having the VANS in downward angles showed a significant increase when compared with the screws in perpendicular angles to the plate (52%), while there was no significant change in the VALS (6%).

The sensitivity analysis showed the relationship between the VANS angles and position used in the models and the total displacement FE results. Increasing the angles in upward or downward directions at the first screw closer to the location where the force was applied resulted in higher displacement (0.46 mm). However, as the screw position moves further away from the applied load and the VANS angle reaches 0°, the displacement decreases to 0.25 mm. On the other hand, the sensitivity analysis of the VALS showed the relationship between the angles and position used in the models and the total displacement results. Increasing the angles in an upward direction at screw one results in higher displacement (0.02 mm) with no noticeable increment in the downward direction. However, as the screw position gets further away from the applied load and the VALS angle decreases, the displacement reduced. The ultimate displacement in the sensitivity analyses for both VALS and VANS corresponds with the upward 40° angle at screw one.

### 4.3. Comparison with Experimental Work

We compared the FEM results with our previous experimental work, where twenty synthetic bones were used. Stiffness and displacement were measured for different screw combinations groups. The displacement results are 52% higher in the experimental work than the FEA. However, the stiffness results were in good agreement with the experimental results. Additionally, the stiffness was the highest when the screws were perpendicular to the plate (0°) and the stiffness decreased as the angle increased to be 40° upward angle. While there was no significant decline in the stiffness as the angle decreased. The displacement increased as the angle upward or downward changed to be the highest at the 40° upward angle.

Our FEA results are consistent with experimental work performed elsewhere [29,30] where VALS with 0–15° angles were recommended in trauma surgery. In one study [29], 2.4 mm locking screws from Depuy Synthes made of stainless steel and titanium-aluminum-niobium-alloy were inserted at angles (0–15°) with 0.8 Nm torque. Results showed that VALS performed better with angles less than 15°. Our FEA of VALC and VANS clearly shows the highest stress at 40° and lowest displacement and stress developed between 0–15°. In another study [30], 5 mm locking screws from Depuy Synthes inserted at angles (0–15°) and recommended minimizing the upward and downward angles would significantly reduce the failure.

In general, the nonunion of the tibiotalocalcaneal fracture was documented after 18 months post-surgery, and this resulted in very high loads on the screws primarily and caused overloading on the plate. Additionally, the investigation suggests that the device underwent damage by pitting corrosion-fatigue mechanism and overloading towards the end to fail the plate and screws in two or more components. Furthermore, the angle of the screw and the distance from loading have significant effects on the plate fixation. In general, both the experimental work and finite element analysis support the assumption that fixing the screws in plates within the angles (0–15°).

## 5. Conclusions

To understand the mechanics of the tibiotalocalcaneal construct made with the PHILOS plating system, qualitative and quantitative analysis methods were applied, and the investigation demonstrated that there are several factors that led to the failure of the PHILOS construct.

1. The presence of surface scratching, inclusions, discoloration, corrosion pits, and beach marks, and cleavage and striations on the fracture surface.
2. The material of construction of the device was governed by ASTM F138-8 or its ISO equivalent 5832 and exhibited inconsistencies in chemistry and hardness requirements.
3. An interaction of S, Ni, and Mo-based inclusions may have raised pitting susceptibility of the PHILOS construct. These features suggest that the device underwent damage by pitting the corrosion-fatigue mechanism and overloading towards the end to fail the plate and screws in two or more components.
4. Upon linking with the screw-holes, a crack, perpendicular to the loading direction also propagated resulting in multiple fractures. The screws failed via the conjoint bending and torsion fatigue mechanisms.
5. VALS and VANS computational simulation illustrated that the stress vs. displacement behavior found to be dependent on where the screws mounted on the plate and having the VANS in downward angles showed a significant increase in maximum stress when compared with the screws in perpendicular angles to the plate, while there was no significant change in the VALS.
6. Both the experimental work and finite element analysis support the assumption that fixing the screws in plates within the angles (<15°) is preferred.

## Figures and Tables

**Table 1:** Stiffness matrix components imported into Ansys.

Elastic Constants	*E_radial_ (GPa)*	*E_circumferential_ (GPa)*	*E_longitudinal_ (GPa)*	*v_radial_*	*v_.circumferential_*	*v_.longitudinal_*	*G_.radial_*	*G_.circumferential_*	*G_.longitudinal_*
Cortical	1.151	1.151	1.894	0.400	0.250	0.250	0.053	0.066	0.061
Trabecular	13.064	13.064	21.294	0.400	0.250	0.250	5.710	7.110	6.580
**Elasticity Tensor Components**	C11	C22	C33	C12	C13	C23	C44	C55	C66
Cortical	1.423	1.483	2.187	0.534	0.297	0.237	0.066	0.061	0.053
Trabecular	16.162	16.835	24.587	6.061	3.367	2.694	7.110	6.580	5.710

**Table 2:** Weibull distribution results.

**Log-likelihood:** 185.16		
**Domain:** 0 < y < Inf		
**Mean:** 0.74913		
**Variance:** 0.0453871		
**Parameter**	**Estimate**	**Std. Err.**
**A**	0.827193	0.0075486
**B**	3.94028	0.0884813
**Estimated covariance of parameter estimates:**		
	**A**	**B**
**A**	5.69813 × 10^−5^	0.000222511
**B**	0.000222511	0.00782895

**Table 3:** The stress intensity factors, maximum von Mises stresses, and fracture toughness for each crack length.

a/2c	$K_{\min}\;(\mathrm{MPa}\sqrt{m})$ (avg ± SD)	$K_{\max}\;(\mathrm{MPa}\sqrt{m})$ (avg ± SD)	$\text{∆}K\;(\mathrm{MPa}\sqrt{m})$ (avg ± SD)	Maximum von Mises Stress (MPa)	316l SS Fracture Toughness (K_1c_) $\left(\mathrm{MPa}\;\sqrt{m}\right)$ [19]
**0.4**	8.12 ± 6.95	72.37 ± 3.21	40.25 ± 5.08	779.93	112
**0.6**	8.44 ± 7.41	76.04 ± 2.18	43.33 ± 4.79	786.31	112
**0.8**	9.37 ± 8.24	86.91 ± 2.41	48.14 ± 5.325	792.47	112
**1**	10.41 ± 9.15	96.52 ± 2.67	53.465 ± 5.91	795.05	112

## Appendix A

**Table A1:** The von Mises stress, displacement, and stiffness for each model (with locking and nonlocking screws) with different angles.

	With Locking Screws			With Nonlocking Screws		
	von Mises Stress (MPa)	Displacement (mm)	Stiffness (N/mm)	von Mises stress (MPa)	Displacement (mm)	Stiffness (N/mm)
**Experimental Study [22]**						
**Group 1 (no locking screws)**	-	-	-	-	0.58	1121.32
**Group 2 (one locking screw)**	-	0.7552	745.3	-	0.7552	745.3
**Group 3 (one locking screw)**	-	0.7746	620.03	-	0.7746	620.03
**Group 4 (two locking screws)**	-	1.2238	1231.23	-	1.2238	1231.23
**Computational Simulation**						
**Perpendicular to the plate (0°)**	37.6020	0.0122	816.7199	93.0720	0.2759	362.3753
**Upward (5°) angle**	38.2771	0.0124	806.5180	94.6232	0.2798	357.4377
**Upward (10°) angle**	45.0284	0.0139	716.9600	110.1352	0.3179	314.5747
**Upward (15°) angle**	48.8468	0.0155	645.6612	155.1200	0.3812	262.3260
**Upward (20°) angle**	53.1884	0.0170	586.9647	164.4272	0.4190	238.4782
**Upward (30°) angle**	59.0027	0.0187	533.6043	173.8895	0.4613	216.7984
**Upward (40°) angle**	67.5130	0.0206	485.0948	184.9030	0.5074	197.0894
**Downward (5°) angle**	38.1834	0.0124	808.6336	94.4109	0.2790	358.2815
**Downward (10°) angle**	43.4163	0.0125	800.6273	95.9621	0.2829	353.4541
**Downward (15°) angle**	44.0914	0.0126	792.7003	133.8900	0.3153	317.1432
**Downward (20°) angle**	50.8427	0.0127	784.8518	143.1972	0.3468	288.3120
**Downward (30°) angle**	54.6611	0.0129	777.0810	152.6595	0.3815	262.1018
**Downward (40°) angle**	58.1430	0.0130	769.3871	163.6730	0.4197	238.2744
